# Effect of Amphipathic HIV Fusion Inhibitor Peptides on POPC and POPC/Cholesterol Membrane Properties: A Molecular Simulation Study

**DOI:** 10.3390/ijms140714724

**Published:** 2013-07-15

**Authors:** António M. T. Martins do Canto, Alfredo J. Palace Carvalho, João P. Prates Ramalho, Luís M. S. Loura

**Affiliations:** 1 Department of Chemistry, School of Science and Technology, University of Évora, Rua Romão Ramalho, 59, 7000-671 Évora, Portugal; E-Mails: canto.antonio@gmail.com (A.M.T.M.C.); ajpalace@uevora.pt (A.J.P.C.); jpcar@uevora.pt (J.P.P.R.); 2 Centre for Chemistry-Évora, Rua Romão Ramalho, 59, 7000-671 Évora, Portugal; 3 Faculty of Pharmacy, University of Coimbra, Health Sciences Campus, Azinhaga de Santa Comba, 3000-548 Coimbra, Portugal; 4 Centre for Chemistry-Coimbra, Rua Larga, 3004-535 Coimbra, Portugal

**Keywords:** AIDS, HIV fusion inhibitor, lipid bilayer, Enfuvirtide, lipid-peptide interaction, molecular dynamics

## Abstract

T-20 and T-1249 fusion inhibitor peptides were shown to interact with 1-palmitoyl-2-oleyl-phosphatidylcholine (POPC) (liquid disordered, ld) and POPC/cholesterol (1:1) (POPC/Chol) (liquid ordered, lo) bilayers, and they do so to different extents. Although they both possess a tryptophan-rich domain (TRD), T-20 lacks a pocket binding domain (PBD), which is present in T-1249. It has been postulated that the PBD domain enhances FI interaction with HIV gp41 protein and with model membranes. Interaction of these fusion inhibitor peptides with both the cell membrane and the viral envelope membrane is important for function, *i.e.*, inhibition of the fusion process. We address this problem with a molecular dynamics approach focusing on lipid properties, trying to ascertain the consequences and the differences in the interaction of T-20 and T-1249 with ld and lo model membranes. T-20 and T-1249 interactions with model membranes are shown to have measurable and different effects on bilayer structural and dynamical parameters. T-1249’s adsorption to the membrane surface has generally a stronger influence in the measured parameters. The presence of both binding domains in T-1249 appears to be paramount to its stronger interaction, and is shown to have a definite importance in membrane properties upon peptide adsorption.

## 1. Introduction

Peptide fusion inhibitors (FI), such as T-20 (also known as Enfuvirtide or Fuzeon) [1] or T-1249 [2] interfere with human immunodeficiency virus (HIV) fusion of the virus envelope with the immune system cell, effectively inhibiting the process (located at the surface of cells and viruses) by binding to the protein machinery responsible by recognition and fusion, namely to the gp41 protein [3–6], the protein responsible for the viral pore formation and membrane fusion [7–9]. In particular, they interfere with the 6-helix bundle (6HB) originating from the interaction of the *C*-terminal heptad repeats (CHR or HR2) with the *N*-terminal heptad repeats (NHR or HR1) of gp41 [7,10].

T-20 is a synthetic 36 amino acid peptide, whose sequence is homologous to the *C*-terminal of HR2 (Heptad Repeat 2) of gp41 [1] and 10 residues from the membrane proximal external domain (MPER), also known as tryptophan-rich domain (TRD), which is the one responsible for the activity of T-20 and is also involved in peptide binding to lipids [11] (this region is, thus, also known as the lipid binding domain, LBD). This first generation peptide is currently one of the more advanced clinical drugs for inhibiting HIV-1 entry and has received fast Food and Drugs Administration (FDA) approval [8,9,11,12]. Despite T-20’s effectiveness, it has already encountered some resistant strains of HIV [9,11,12]. T-1249 is a synthetic 39 amino acid peptide composed of sequences derived from HIV-1, HIV-2 and simian immunodeficiency virus (SIV) [2]. This peptide also possesses a TRD domain at the *C*-terminal, but it has an additional gp41 functional domain known as the pocket binding domain (PBD). This amphipathic *N*-terminal domain is responsible for efficient binding of FIs to the deep pockets formed by gp41 NHR trimers [13]. This fusion inhibitor has been shown as a stronger inhibitor of HIV entry than T-20, and it retains function against T-20 resistant strains [9,11,12], inhibiting 6HB formation more efficiently [14].

Peptides designed to perform this way are developed to be amphipathic and, thus, to be able to interact effectively with both the protein target, water and, also, the membranes [15,16]. Interaction of T-20 and T-1249 has been addressed with both experimental and simulation approaches [15–20]. It was observed that both peptides interact with 1-palmitoyl-2-oleyl-phosphatidylcholine (POPC) (liquid disordered, ld) and POPC/cholesterol (1:1) (POPC/Chol) (liquid ordered, lo) bilayers [15–20]. Experimentally, T-1249 was shown to interact more effectively with both membranes, namely with the POPC/Chol membranes with which T-20 was shown experimentally to have no detectable interaction [17]. A molecular dynamics approach to the same system showed interaction between T-20 and POPC/Chol membranes, but much weaker and ineffective than that observed with T-1249 [18–20]. As such, a working model for these peptides was suggested, postulating that the interaction/adsorption of these peptides with/to the membranes was paramount for function. In fact, effective adsorption to the membranes of both the viral envelope and the immune system cells increases the local concentration of the fusion inhibitors and, thus, improves their availability, maximizing their effectiveness [17]. Peptides like these should be also able to interact with Chol-rich bilayers, such as the ones of the viral envelope [19–21]. All this has been supported by the fact that T-1249 interacts effectively with Chol-containing membranes, requires only a daily dose to be effective and also retains effectiveness against T-20 resistant strains of HIV [9,11,12,15].

Despite recent works, a complete and detailed molecular picture of the inhibitory mechanism promoted by these molecules and of the role of the membranes in it is still lacking. A molecular dynamics (MD) approach is used here to evaluate the behavior of membranes in the ld (POPC) and lo (POPC/Chol 1:1) phases upon peptide fusion inhibitor interaction. To this effect, parameters, such as H bond formation between membrane lipids and water (Sol), lateral diffusion coefficients (*D*_lat_) of the lipids under scrutiny, rotational dynamics of selected molecular axes and overall acyl chain order parameters, are calculated and discussed.

## 2. Results and Discussion

### 2.1. Equilibration of the Membrane System, Cross-Sectional Area per Lipid and Membrane Thickness

**Figure 1:**
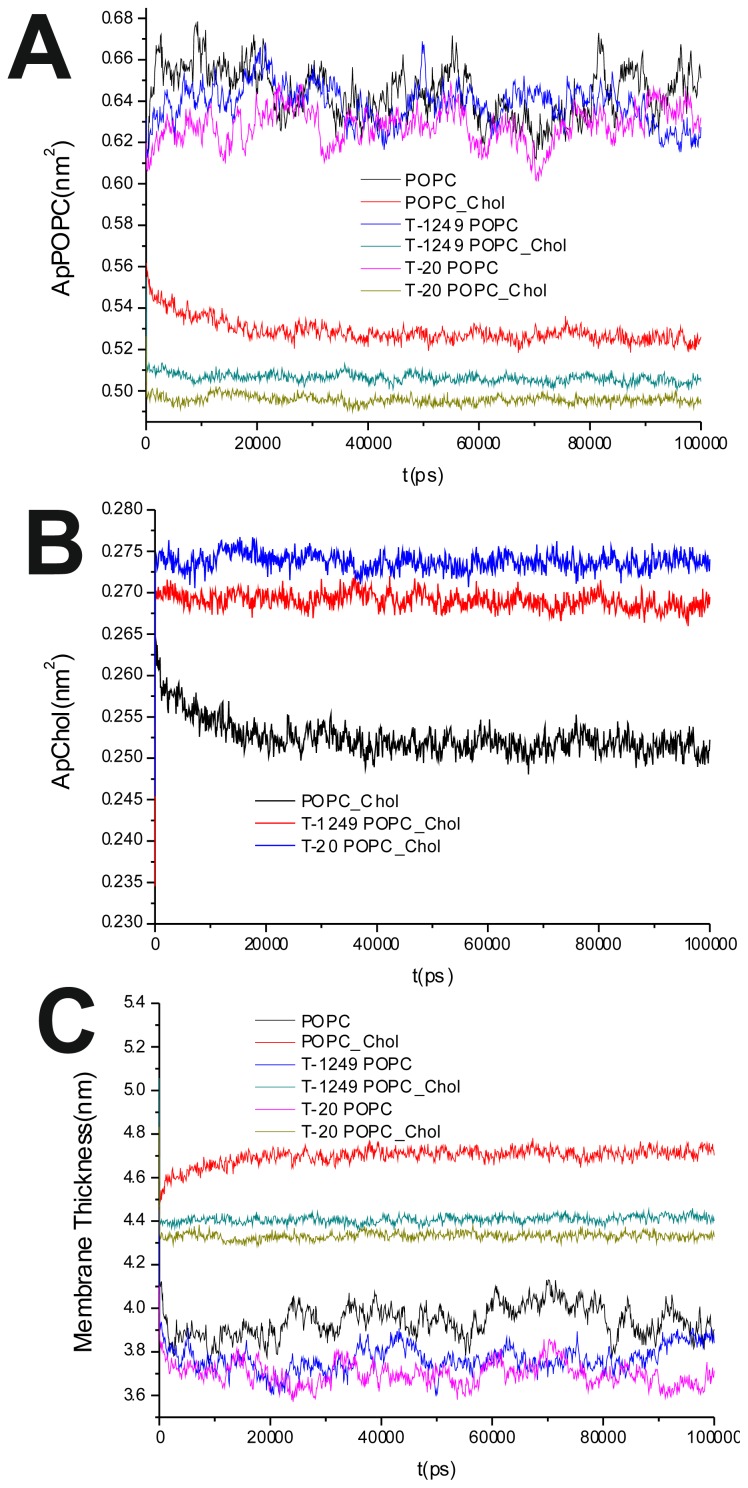
(**A**) Area per 1-palmitoyl-2-oleyl-phosphatidylcholine (POPC) time course; (**B**) Area per cholesterol (Chol) time course; (**C**) Membrane thickness time course.

To evaluate the process of the systems’ equilibration, time profiles of the surface area/POPC (Figure 1A), surface area/Chol (Figure 1B) and membrane thickness were calculated as in [22] [Equations (1) and (2)] and recorded for the production simulation (100 ns):

(1) $$ApPOPC=\frac{2A_{box}}{V_{box}-N_{W}V_{W}}\left[\frac{V_{box}-N_{W}V_{W}-xN_{lipid}V_{Chol}-V_{peptide}}{(1-x)N_{lipid}}\right]$$

(2) $$ApCHOL=\frac{2A_{box}V_{Chol}}{V_{box}-N_{W}V_{W}-V_{peptide}}$$

In these equations, *ApPOPC* is the cross-sectional area per POPC molecule, *ApCHOL* is the cross-sectional area per Chol molecule, *A*_box_ is the area of the *xy* plane of the simulation box, *V*_box_ is the simulation box total volume, *N*_w_ is the number of water molecules, *V*_w_ is the volume of the water molecule, *x* = 0.00 or 0.50 is the Chol mole fraction, *N*_lipid_ is the number of lipid molecules, *V*_Chol_ is the volume of the Chol molecule [22] and *V*_peptide_ is the volume of the peptide molecule, determined from the peptide simulation in water by averaging *V*_peptide_ = *V*_box_ − *N*_w_ × *V*_w_ for the last 25 ns of the simulation.

The average of the surface area per lipid was stable over the final 80 ns of the simulation, which led us to the conclusion that the simulated systems had reached a steady state after 20 ns of simulation (Figure 1A,B). This was also verified by observation of the membrane thickness parameter. Membrane thickness was determined by the ensemble average of the P–P distance between phosphate P atoms of opposite leaflets. Figure 1C shows the time variation of the latter, and as can be observed, stabilization is achieved and maintained over the last 80 ns of the simulation.

It has been shown that the cross-sectional area per POPC decreases, whereas the cross-sectional area per Chol increases, in all systems upon peptide adsorption (Table 1). This suggests that peptide adsorption would have a condensation-like effect on the bilayers, but also that Chol molecules in the POPC/Chol bilayers are more exposed to solvent molecules in membranes interacting with either T-20 or T-1249. Peptide adsorption has little or no effect in the membrane thickness (Table 1) of POPC bilayers (the effect appears to be always a decrease, larger for T-20) and induces a decrease in the membrane thickness of POPC/Chol bilayers (here, too, the effect is greater for T-20). In these membranes, the free volume is low, so if the peptides are inducing decreases in both the cross-sectional area per POPC and the membrane thickness, this could account for the exposure of the Chol molecules to water, reducing the umbrella effect that protects and stabilizes Chol molecules in these bilayers [23].

### 2.2. H Bonds between POPC, Chol and Sol Molecules

Formation of H-bonds between POPC, Chol and water molecules was investigated. For this analysis, an H-bond for a given donor—the H-acceptor triad was registered each time—the donor acceptor distance was <0.35 nm and the H-donor-acceptor angle was <30°.

Table 2 shows the average number of H-bonds formed between the three groups under scrutiny (POPC, Chol and Sol—solvent, *i.e.*, water). Both peptides when interacting with the model bilayers cause a decrease in the number of H bonds formed between POPC and solvent molecules. This effect is generally more pronounced in the T-1249 systems, namely in the T-1249/POPC system, where the decrease is greater. Behind this effect appears to be the fact that T-1249 is able to interact more strongly via H bonds with the bilayer lipids than T-20 [19,20], involving more lipid molecules that would, otherwise, be available for interaction with the solvent molecules. This observation is also supported by the fact that the decrease in POPC-Sol H bonds is always greater in the top bilayer, suggesting the peptide adsorption as a direct cause to this effect. Peptide interaction also causes a decrease in the Chol-water H bond number, but this decrease is so small, that it appears to be due mainly to a reduction in the number of Chol molecules accessible to the water molecules (because they are covered by the adsorbing peptide) and, thus, capable of linking via H bonds. Finally, the interaction of both peptides with POPC/Chol bilayers induces a small increase in the number of H bonds between POPC and Chol molecules, apparently caused by the tighter packing of the bilayer lipids, due to the peptide adsorption to the top leaflet that pushes [19,20], as reported earlier, the top leaflet bilayer lipids (located closer to the peptide) towards the hydrophobic core.

In order to evaluate H bond dynamics, the H bond’s lifetime was calculated as described in the GROMACS manual [24]. In short, the lifetime of the H-bonds, shown on Table 3, is calculated from the average over all autocorrelation functions of the existence functions (either zero or one) of all H-bonds:

(3) $$C(\tau )=\langle S_{i}(t)Si(t+\tau )\rangle$$

with *S_i_*(*t*) ɛ {0; 1} for an H-bond, *i*, at time, *t*. The integral of *C*(τ) then gives an estimate of the average H-bond lifetime, τ_HB_:

(4) $$\tau_{HB}=\int_{0}^{\infty }C(\tau )d\tau$$

Upon peptide adsorption, τ_HB_ for the POPC-Sol H bonds increases in the top leaflet of the POPC bilayers under scrutiny (Table 3). In the bottom leaflet, τ_HB_ for POPC-Sol H bonds remains mostly unaltered. Peptide adsorption creates a burrow-like crater in the POPC bilayers [19,20], further exposing the POPC molecules of the crater’s rim. This exposure to the Sol molecules may be responsible for the increase in POPC-Sol H bond lifetime; the effect is local, and hence, the average is only slightly increased. In POPC/Chol bilayers, which are significantly more rigid and, therefore, do not allow for an exposure in the same order as in the POPC bilayers [19,20], the effect is virtually insignificant.

Peptide adsorption also evokes an increase in the Chol-Sol and POPC-Chol H bond lifetimes, especially in the latter. In the Chol-Sol H bonds, the increase is small (and smaller in the T-1249/POPC/Chol system), and it may be caused by the increased exposure of Chol molecules to Sol: cross-sectional area per Chol increases upon peptide adsorption (also less in the T-1249/POPC/Chol system). This further exposure to Sol molecules would facilitate H bond formation and persistence. As T-1249 interacts with Chol via H bonds [20], this effect is lessened.

POPC-Chol H bond lifetimes are significantly increased upon peptide adsorption. The effect has a greater magnitude in the top bilayer of both peptide containing systems. Peptide adsorption induces pressure on the bilayer surface. This pressure pushes one leaflet against each other (in the *z* direction), but also, the molecules against each other in the *xy* plane. This increased proximity facilitates H bond formation and persistence. POPC/Chol membranes have very little free volume and slow dynamics (much slower that POPC membranes), and as such, effects as these should propagate easily inside the bilayer, thus causing the POPC-Chol H bonds to have longer lifetimes. Nonetheless this effect appears to be dependent on the distance to the peptide, since it is, in both cases, weaker in the bottom monolayer.

### 2.3. Interaction Energies between TRD and PBD and the Bilayer Components

We have previously reported the importance of Lennard–Jones (LJ) and Coulomb interactions (between the peptides and the membrane lipids) as the driving force in the peptide behavior towards the membranes: first leading to a decrease of the distance between the peptides and the membrane surface and subsequently favoring the adsorption of the peptides to the membrane surface [20]. Due to the apparent importance of the amphipathic domains, TRD and PBD, in the peptides’ function, interaction energies between these domains and the membrane lipids in each system were calculated as shown in Table 4. In the pure POPC systems, the T-1249’s TRD domain has higher interaction energies in all counts, and its PBD interaction energies further enhance the peptide’s capability to approach and interact with the membrane. However, it is in the POPC/Chol systems that the relevance of the PBD domain really stands out. Although the T-1249’s TRD domain has lower interaction energies with the membrane lipids, the existence of the PBD domain balances that effect and causes the peptide to have higher LJ interaction energies (view sum column) and close to no changes in the Coulomb interaction energies.

### 2.4. Lateral Diffusion of POPC and Chol

The lateral diffusion coefficients, *D*_lat_, of the lipids were calculated from the two-dimensional mean square displacement (MSD) using the Einstein relation:

(5) $$D_{lat}=\frac{1}{4}\underset{t\to \infty }{\text{lim}}\frac{dMSD(t)}{dt}$$

in which *MSD* (*t*) can be defined as:

(6) $$MSD\;(t)=<{\left\|\vec{R_{\iota }}(t+t_{0})-\vec{R_{\iota }}(t_{0})\right\|}^{2}>$$

Where $\vec{R_{\iota }}$ is the (*x*,*y*) position of the center of mass of the molecule, *i*, of a given species, and the averaging is carried out over all molecules of this kind and time origins. To eliminate noise due to fluctuations in the center of mass of each leaflet, all lipid *MSD* analyses were carried out using trajectories with a fixed center of mass of one of the leaflets [25,26]. MSD and respective *D*_lat_ were calculated for POPC and Chol as the average of both leaflets and for the leaflets separately.

Table 5 shows *D*_lat_ for the molecules under scrutiny, POPC and Chol, in all systems under study. In the POPC bilayer systems, peptide adsorption induces a decrease in POPC’s *D*_lat_ more pronounced in the top bilayer and for the T-1249 peptide’s adsorption. Adsorption may be causing a slowing of the membrane diffusion dynamics. In the T-20/POPC/Chol system, peptide interaction has the opposite effect; an increase in *D*_lat_. T-20, for most of the simulation run, only interacts with the POPC/Chol bilayer via the *C*-terminus amino acids, leaving the rest of the helix with a high rotational and translational freedom [19] and, also, a high *D*_lat_[19]. This mobility may be thus influencing POPC’s diffusion dynamics, increasing its *D*_lat_. In the T-1249/POPC/Chol system, the POPC’s *D*_lat_ in the top leaflet of this system decreases. T-1249 interacts more strongly with the bilayers than T-20 [18–20], and so, the effect observed for the POPC bilayers appears to be maintained in this system. Chol molecules show an increase in *D*_lat_ in all peptide-containing systems. This increase may be a result of the disordering caused by the peptides’ adsorption (see Section 2.6., Tables 6 and 7). The smaller increase is in the top leaflet’s Chol of the system containing T-1249, and that may be caused by the fact that Chol interacts more strongly with T-1249 (via H bonds) than with T-20 (which showed no such interaction) [19].

### 2.5. Rotational Dynamics of Selected Axis of Membrane Lipids

The rotational dynamics of several axes (POPC P–N axis (P8→N4), *sn*-1 long axis (C2→C15), *sn*-2 long axis (C2→C17) and Chol long axis (C2→C20)) was studied. In this way, motions both in the headgroup and in the hydrophobic regions were addressed. To this end, rotational auto correlation functions C(*t*), defined in Equation 7, were calculated.

(7) $$C(t)=<P_{2}\left(\cos\theta (\zeta )\right)>$$

Here, θ(ζ), for the sake of commodity, is the angle between a vector defined in the molecular framework at times, ζ and t + ζ, and P_2_(x) = (3x^2^ − 1)/2 is the second order Legendre polynomial. Averaging is performed over ζ, which, assuming a sufficiently ergodic trajectory, is an approximation of the ensemble average. All calculated C(t) functions decay to a residual value, C_∞_ > 0, probably denoting a hindered rotation [26,27].

**Figure 2:**
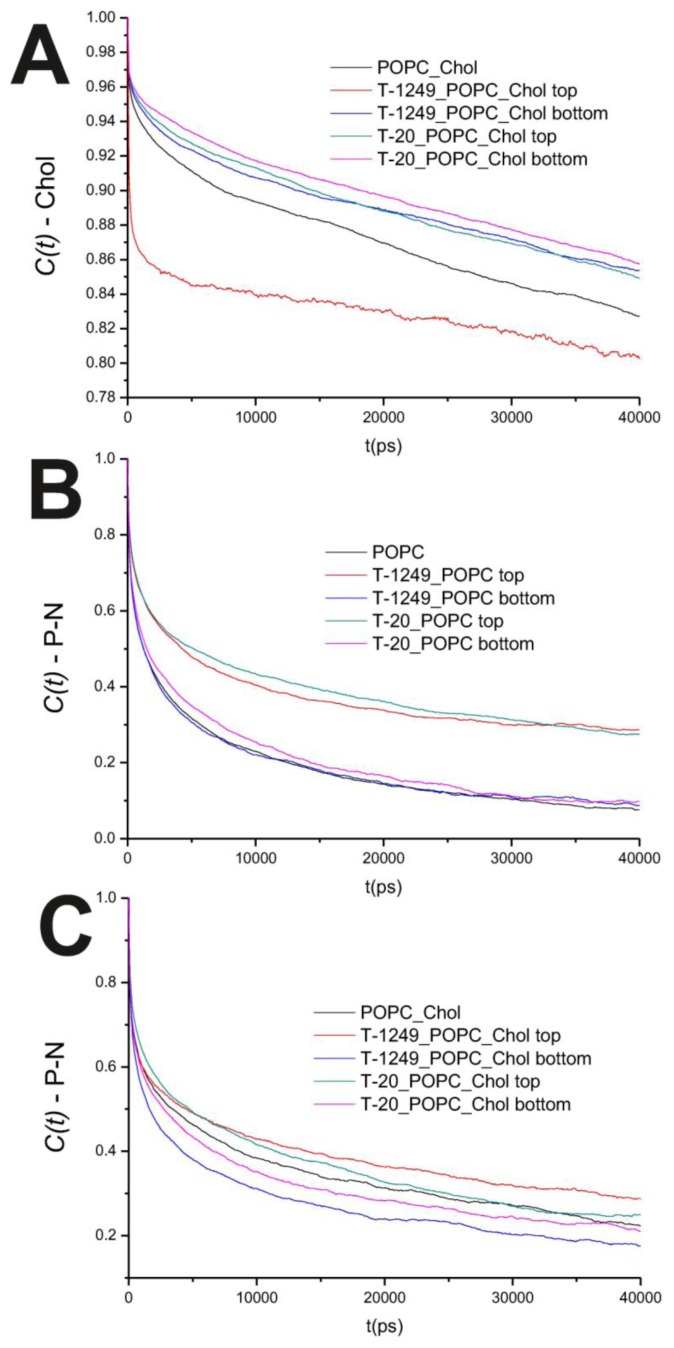
(**A**) Auto correlation functions for the Chol axis in the POPC/Chol bilayer systems; (**B**) Auto correlation functions for the P–N axis in the POPC bilayer systems; (**C**) Auto correlation functions for the P–N axis in the POPC/Chol bilayer systems.

**Figure 3:**
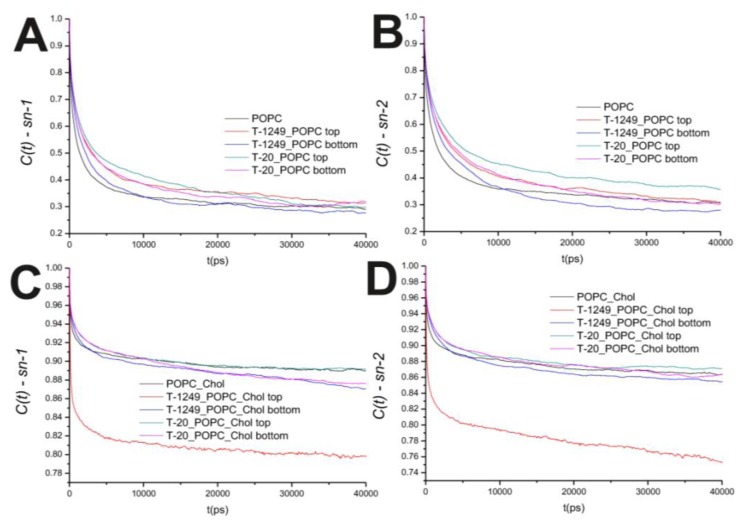
(**A**) Auto correlation functions for the *sn-1* axis in the POPC bilayer systems; (**B**) Auto correlation functions for the *sn-2* axis in the POPC bilayer systems; (**C**) Auto correlation functions for the *sn-1* axis in the POPC/Chol bilayer systems; (**D**) Auto correlation functions for the *sn-2* axis in the POPC/Chol bilayer systems.

In the POPC bilayer systems, peptide interaction with the model membranes causes an increased hindrance to rotational movements in the P–N axis (Figure 2B). This is mostly restricted to the top leaflet, which suggests that peptide adsorption and interaction with POPC molecules slows P–N axis rotational movement at the interface. However, this is not restricted to the interface, and a similar effect is verified in both the *sn-1* and *sn-2* axis located in the hydrophobic core (Figure 3). In the POPC/Chol systems, peptide adsorption has almost no effect in the dynamics of the *sn*-1 and *sn*-2 axes in both leaflets, with the exception of the top leaflet interacting with T-1249, in which both axes show less restricted rotation, *i.e.*, tumbling of the rotational autocorrelation function to lower values (Figure 3C,D). In these systems, the dynamics of the P–N axis is slowed for the top leaflets and faster in the bottom leaflets; this effect is of greater magnitude in the T-1249 adsorption, which implies direct correlation to the degree of interaction of the peptide with the membrane (Figure 3C). Chol dynamics is generally slowed upon peptide adsorption in both leaflets in the T-20 system and in the bottom leaflet in the T-1249 system (Figure 3A). Chol shows a faster dynamics in the top leaflet of the T-1249/POPC/Chol system, and this appears to be a similar phenomenon, as in the acyl chain axis. Since Chol interacts strongly with T-1249, namely via H bonds [20], and because T-1249 has a diffusion dynamics faster than the bilayer lipids [20], it induces Chol to assume a faster dynamics also, interfering, thus, with the bilayer core dynamics.

### 2.6. Order Parameters

The order parameter tensor, *S*, is defined as:

(8) $$S_{ab}=\frac{1}{2}<3\;\text{cos}(\theta_{a})\;\text{cos}(\theta_{b})-\delta_{ab}>\;a,b=x,y,z$$

where θ_a_ (or α_b_) is the angle made by a^th^ (or b^th^) molecular axis with the bilayer normal and δ_ab_ is the Kronecker delta (< > denotes both ensemble and time averaging). In our simulations, using a united atom force field, the order parameter for saturated and unsaturated carbons, −*S*_CD_, can be determined using the following relations [28]:

(9) $$-S_{CD}^{Sat}=\frac{2}{3}S_{xx}+\frac{1}{3}S_{yy}$$

(10) $$-S_{CD}^{Unsat}=\frac{1}{4}S_{zz}+\frac{3}{4}S_{yy}+\frac{\sqrt{3}}{2}S_{xy}$$

−*S*_CD_ may vary between 0.5 (full order along the bilayer normal) and −0.25 (full order along the bilayer plane), whereas −*S*_CD_ = 0 denotes isotropic orientation. Due to the slow convergence of this parameter [27], analysis was restricted to the last 50 ns of the simulations. For an immediate comparison, −*S*_CD_ ensemble averages for the last 50 ns of each simulation, along the *sn*-1 chains, are shown in Table 6 for the systems under study.

In the 50 ns time scale, T-1249 has a larger effect in the −*S*_CD_, on both POPC and POPC/Chol bilayers. T-1249 was shown to be able to interact in a stronger manner with the model membranes [19,20]; both its Lennard-Jones and Coulomb interaction energies (T-1249-POPC) are higher than T-20’s [20]. Additionally, it forms more H bonds with the bilayer’s POPC than T-20, and the bonds are distributed more evenly throughout the length of the peptide helix [20], apparently stabilizing this interaction in a more effective way. T-20 and T-1249 have opposite overall average effects in the −*S*_CD_ of the *sn-*1 acyl chain of the POPC bilayer, while T-1249 decreases the average of −*S*_CD_, T-20 increases this parameter, albeit by a small amount. This stronger interaction may account for the influence it has on *sn-*1. T-1249 has a fast (faster than POPC’s) diffusion dynamics [19] and is able to, nonetheless, form H bonds with POPC, thus dragging POPC molecules in its movement. This enhanced mobility should reflect in a disordering of the acyl chains and account for the lowering of −*S*_CD_. The same phenomenon appears to be responsible for the lowering of −*S*_CD_ in the POPC/Chol bilayers. These bilayers are in the lo phase, highly ordered and with a slow diffusional dynamics. Upon T-20 and T-1249 interaction with these bilayers, −*S*_CD_ decreases (Table 6). The peptides have faster dynamics than the bilayer molecules and are able to form H bonds with POPC (and Chol in the T-1249’s case) [18–20], thus “dragging” POPC or Chol molecules faster than the surrounding ones that are not linked to the peptide and, thus, interfering with the highly ordered structure of the bilayer. This effect is more pronounced in the T-1249 adsorption. The fact that its interaction with membrane lipids is stronger (and the only peptide that forms H bonds with Chol) [20] appears to be a significant influence in the lowering of −*S*_CD_.

**Figure 4:**
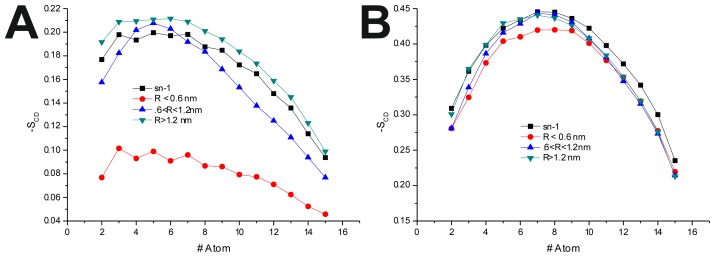

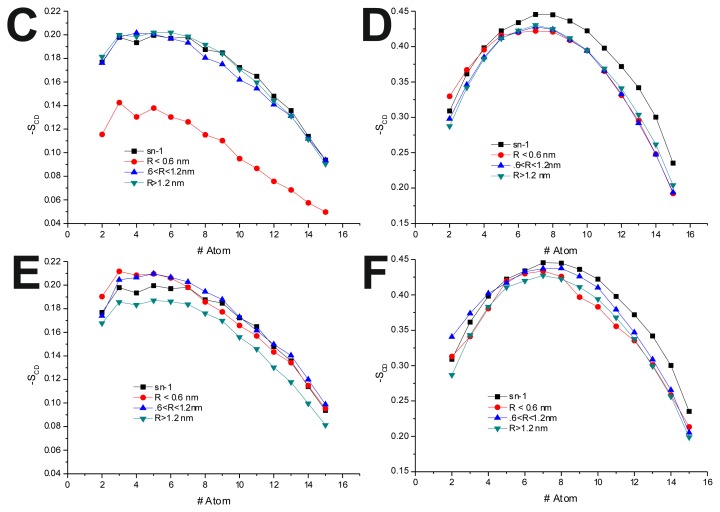
−*S*_CD_(*R*) for the *sn-*1 acyl chain. *R* is the distance separating the peptide’s TRD (**A**,**B**,**C**,**E**) and PBD (**D**,**F**) from each individual POPC molecule. All systems were thus analyzed: (**A**) T-20 + POPC; (**B**) T-20 + POPC/Chol; (**C**) T-1249 + POPC; (**D**) T-1249 + POPC/Chol; (**E**) T-1249 + POPC; and (**F**) T-1249 + POPC/Chol. In all graphics, *sn-*1 represents the −*S*_CD_ values for the *sn-*1 acyl chain in the equivalent system (POPC or POPC/Chol, whichever is relevant), but in the absence of peptide.

To further investigate the effect of the peptide adsorption to the membrane surface on the −*S*_CD_, these parameters were calculated as a function of the minimal atomic distance (for three different distance bins: *R* < 0.6 nm, 0.6 nm < *R* < 1.2 nm and *R* > 1.2 nm, also, in the last 50 ns of each production run) between the peptide’s TRD and PBD domains and each individual POPC molecule (Figure 4 and Table 6). In the disordered POPC systems, proximity to the TRD (*R* < 1.2 nm) causes a decrease in −*S*_CD_ in the *sn-*1 of the POPC molecules closer to the TRD domain (Figure 4A,C and Table 7), this effect being stronger in the T-20-containing system. In the T-1249 + POPC system, PBD domain proximity (*R* < 1.2 nm) causes an increase in −*S*_CD_ of the *sn-*1 acyl chain (Figure 4E). In this system, for *R* > 1.2 nm, the effects of interaction with both TRD and PBD domains, in both cases, are reversed from those described above, which appears to be due to the interaction of the POPC molecules with the opposite domain of the peptide (Figure 4 and Table 7). T-20, having no PBD domain, suffers no such effect and, as such, imposes a stronger decrease in −*S*_CD_ of the *sn-*1 acyl chain. The localized compression of T-20 induces around its TRD, resulting from the way it burrows itself in the membrane interface and compresses the POPC molecules, both in *z* direction and the *xy* plane, and the latter may be responsible for the increase in −*S*_CD_ of the *sn-1* acyl chain observed for *R* > 1.2 nm.

In the POPC/Chol, lo, systems, interaction with the TRD and PBD domains causes an overall decrease in −*S*_CD_ on all bins (Figure 4B,D,F and Table 7). In the T-20 + POPC/Chol system, this effect decreases with the distance from the TRD domain, whereas in the T-1249 + POPC/Chol system, there is almost no variation from bin to bin (Figure 4B,D and Table 7). A similar observation can be made for the PBD effect on −*S*_CD_, in which the decrease in −*S*_CD_ is similar in the closer and outer bin, being smaller in the middle bin (Figure 4F and Table 7). These membranes are in a highly ordered state, and the interaction of these peptides that diffuse more rapidly than the lipids with which they interact, as described earlier, causes a disordering effect in the bilayer. This effect is stronger in the T-1249-containing systems, as it interacts more strongly with POPC/Chol bilayers.

## 3. Simulation and Analysis Details

**Figure 5:**
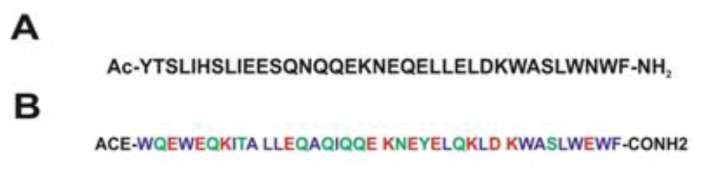

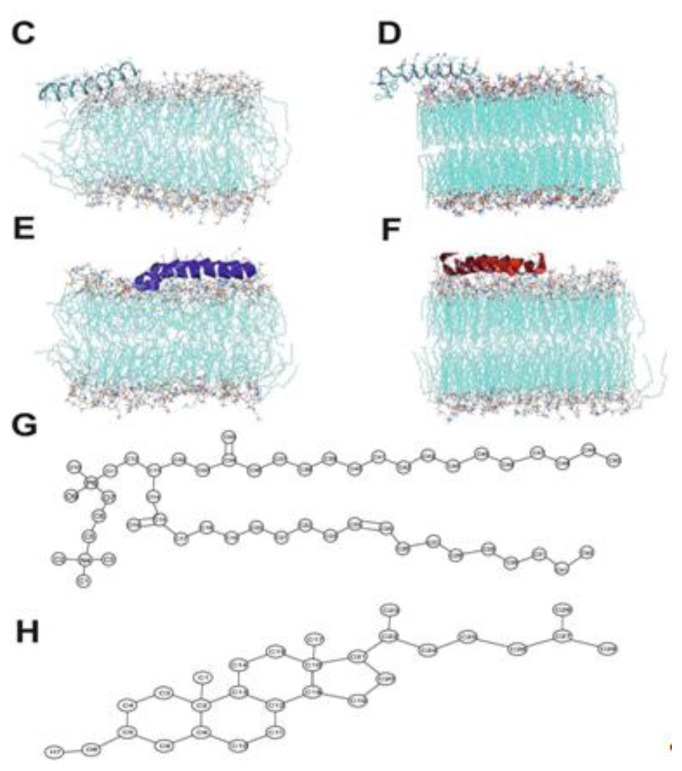
(**A**) T-20 amino acid sequence; (**B**) T-1249 amino acid sequence; (**C**) T-20/POPC final structure snapshot; (**D**) T-20/POPC/Chol final structure snapshot; (**E**) T-1249/POPC final structure snapshot; (**F**) T-1249/POPC/Chol final structure snapshot; (**G**) POPC structure and atom numbering; (**H**) Cholesterol structure and atom numbering. Adapted from [18,19].

The initial α-helix model of the peptides (T-20 and T-1249) (see Figure 5A,B for the primary structure) was built with the Arguslab 4.01 package [29] (both peptides were modeled at pH = 7) and solvated in a cubic simulation box with SPC water [30], allowing for a distance between the peptide and the box walls of 0.5 nm. POPC model molecules (Figure 5G) and their bonded and non-bonded parameters were downloaded from the Tieleman group web page [31]. Cholesterol model molecules (Figure 5H) and their bonded and non-bonded parameters were taken from Holtje *et al.* [32] and were downloaded from the GROMACS web page [33]. Initial models of both membranes (POPC, 126 molecules, and POPC/Chol (1:1), 240 molecules in total; see Figure 5C–F) were built. To this purpose, one POPC molecule (with mostly stretched and parallel acyl chains) from the original POPC bilayer pdb file (together with one Chol molecule in the case of the POPC/Chol system) was used to build custom size model bilayers using GROMACS model preparation packages [34,35]. The latter was also used to perform all simulations. The GROMACS force field (which is a modified GROMOS87 force field) was used to describe all the interactions (see the GROMACS manual for details [24]. Molecular dynamics of these systems, under the same conditions as the final MD runs (see below), were performed for at least 50 ns to ensure that the bilayers were equilibrated prior to the peptide inclusion in the system. Peptide and bilayer models were then combined, and the final structure obtained after 100 ns simulation of each peptide in water was used as the initial structure of the simulations of the peptide interacting with the bilayer systems. The zz dimension of the simulation box was increased for this purpose, and the peptide molecule was positioned, with the helix’s axis parallel to the bilayer surface (but, otherwise, random orientation of its residues relative to the bilayer), at a distance of at least 2.0 nm above the average position of the lipid P atoms of the top leaflet. The number of added SPC water molecules was sufficient to ensure full peptide and bilayer hydration in all systems (9602 water molecules added to the POPC bilayer system—with average dimensions of 6.4 × 6.1 × 11.4 nm^3^ and 7398 water molecules added to the POPC/Chol bilayer system—with average dimensions of 6.7 × 6.9 × 9.4 nm^3^). Systems with no added peptide were also simulated, and the main structural lipid properties were successfully verified for validation purposes. Prior to the production MD simulation, all systems underwent a steepest-descent energy minimization of the structure followed by a small MD run to properly allow the solvent molecules to adjust/relax around the peptide or membrane. Extensive MD simulations were then performed under constant number of particles, pressure (1 bar) and temperature (300 K) and using periodic boundary conditions. Pressure and temperature control was carried out using the weak-coupling Berendsen schemes [36], with coupling times of 1.0 ps and 0.1 ps, respectively. Isotropic pressure coupling was used for the peptides simulation in water, and semiisotropic pressure coupling was used in all the other simulations. All bonds were constrained to their equilibrium values using the SETTLE (analytical version of the SHAKE and RATTLE algorithm for rigid water models) algorithm [37] for water and the LINCS (linear constraint solver) algorithm [38] for all other bonds.

The systems were simulated for 100 ns, with a time step of 2 fs. The long-range electrostatic interactions were calculated by the particle-mesh Ewald (PME) summation method [39]. A cut-off of 1.0 nm was used for both van der Waals and the PME direct-space component of electrostatic interactions. Analyses were carried out, mostly using the GROMACS 3.3.3 analysis package [34,35]. Errors were calculated according to the block method of Flyvbjerg and Petersen [40], except for the diffusion coefficients, whose errors were estimated by calculating the difference between the values calculated from the two halves of the sampling interval.

## 4. Conclusions

100 ns molecular dynamics simulations of solvated bilayers (POPC—in the liquid disordered phase and POPC/Chol 1:1—in the liquid ordered phase) were performed for comparison purposes. Those bilayers were also analyzed and several parameters where determined, both for validation purposes and comparison with the peptide simulations. In this work, bilayers in two different phases were subject to interaction with two peptide HIV fusion inhibitors: T-20, also known as Enfuvirtide, or Fuzeon, already approved and in use in AIDS therapeutics [11], and T-1249, a second generation peptide known to be more effective than the former [9,11,12,15].

Our results show that, upon peptide adsorption, the bilayers behave differently and suffer sizeable structural and dynamical modifications. As reported elsewhere [17,19], T-20 interacts poorly, by comparison with T-1249 [17,20], with membrane models both in the ld and lo phases.

The ability to form H bonds with the bilayers appears to be paramount in peptide-bilayer effective interaction: not only T-1249 forms more H bonds with both studied bilayer models than T-20 (≈13% in POPC bilayers and ≈21% in POPC/Chol bilayers [19,20]), but also, the amino acid residues responsible for those H bonds span the length of the peptide helix [19,20]. The amino acid residues responsible for H bond forming in T-20 are mostly restricted to the *C*-terminus TRD, whereas in T-1249, the PBD domain is also responsible for forming H bonds with the model membranes [19,20]. These results concur with recent experimental observations that show a higher affinity for the interaction of T-1249 with membranes in ld and lo phases [41]. When interacting with a POPC bilayer, T-1249 is able to stabilize the binding to the membrane by inducing an overall disordering of the membrane, a phenomenon that does not appear to have correspondence in T-20’s behavior. Although T-20 is capable of adsorption to the POPC bilayer, its interaction causes an overall ordering of the bilayer that is only an average effect, since in the vicinity of T-20’s TRD, the decrease in −*S*_CD_ of the *sn-1* acyl chain is the largest observed in this work. When these peptides interact with the highly ordered POPC/Chol bilayers, both peptides cause an overall disordering of the bilayers. However, T-1249’s ability to form H bonds stabilizes the adsorption, thus disordering the bilayer more efficiently.

These two peptides are very similar, having, generally, a large deal of homology between them. The main difference between them is the existence of a PBD in T-1249, both having a TRD. Our own previous results have demonstrated that in both bilayer systems, PBD is responsible for a high number of the peptide’s H bonds, namely ≈23% and ≈12% of total H bonds formed between the peptide and the bilayer in the POPC and POPC/Chol systems, respectively [19,20]. The importance of a PBD, not only in the interaction of fusion inhibitor peptides with gp41, but also with membranes, has been suggested by studies that showed effective binding to membranes of compounds capable of effectively binding to the hydrophobic pocket, that is, the target of the PBD [19,20,42–45]. As such, a putative role of the PBD in the modulation of the peptide interaction with the bilayers has been previously suggested [41]. Our results show an atomic-scale perspective of that modulation via H bond formation, namely, with Chol in the POPC/Chol system, and reinforce the postulate that the development of the fusion inhibitor possessing both TRD and PBD should be pursued for enhanced effectiveness, both in the binding to gp41 and membranes [13,41]. A more stable adsorption to bilayers in both phases would allow for a local (in the vicinity of both the cell membrane and the viral envelope) higher concentration of the HIV fusion inhibitors, and thus, a more effective action, supporting the working model cited above [15,17]. With this in mind, further developments of fusion inhibitor peptides should also focus on membrane interaction as a mean to improve their molecular targeting properties, and thus, their efficiency.

## Figures and Tables

**Table 1:** Cross-sectional area per (Ap) lipid in all systems under study and their respective membrane thickness.

System	Ap POPC (nm^2^)	Ap Chol (nm^2^)	MT (nm)
POPC	0.645 ± 0.020		3.82 ± 0.13
POPC/Chol	0.526 ± 0.017	0.252 ± 0.008	4.59 ± 0.14
T-20 + POPC	0.633 ± 0.020		3.79 ± 0.13
T-20 + POPC/Chol	0.495 ± 0.016	0.274 ± 0.009	4.32 ± 0.14
T-1249 + POPC	0.634 ± 0.009		3.80 ± 0.05
T-1249 + POPC/Chol	0.505 ± 0.002	0.269 ± 0.001	4.43 ± 0.11

**Table 2:** Average number of H bonds between membrane lipids and water. Sol, solvent.

System	Leaflet	H Bond number/lipid		

		POPC-Sol	Chol-Sol	POPC-Chol
POPC	*Both*	6.47 ± 0.01		

POPC/Chol	*Both*	6.30 ± 0.02	0.82 ± 0.01	0.66 ± 0.01

T-20 + POPC	*Both*	6.37 ± 0.01		
	*Top*	6.30 ± 0.03		
	*Bottom*	6.43 ± 0.02		

T-20 + POPC/Chol	*Both*	6.17 ± 0.02	0.70 ± 0.01	0.77 ± 0.01
	*Top*	6.12 ± 0.02	0.70 ± 0.01	0.74 ± 0.01
	*Bottom*	6.22 ± 0.02	0.71 ± 0.01	0.80 ± 0.01

T-1249 + POPC	*Both*	4.84 ± 0.02		
	*Top*	4.77 ± 0.02		
	*Bottom*	4.92 ± 0.04		

T-1249 +POPC/Chol	*Both*	6.13 ± 0.01	0.72 ± 0.01	0.79 ± 0.01
	*Top*	5.98 ± 0.01	0.72 ± 0.01	0.77 ± 0.01
	*Bottom*	6.28 ± 0.01	0.72 ± 0.01	0.82 ± 0.01

**Table 3:** H bond lifetimes for H bonds formed between bilayer lipids and water.

System	τ_HB_ (ns)		

	POPC-Sol	Chol-Sol	POPC-Chol
POPC	0.10 ± 0.01		
POPC/Chol	0.08 ± 0.01	0.11 ± 0.01	25.03 ± 0.01
T-20 + POPC top	0.16 ± 0.01		
T-20 + POPC bottom	0.11 ± 0.01		
T-20 + POPC/Chol top	0.11 ± 0.01	0.16 ± 0.01	38.33 ± 0.01
T-20 + POPC/Chol bottom	0.09 ± 0.01	0.14 ± 0.01	34.70 ± 0.01
T-1249 + POPC top	0.16 ± 0.01		
T-1249 + POPC bottom	0.10 ± 0.01		
T-1249 + POPC/Chol top	0.10 ± 0.01	0.14 ± 0.01	39.05 ± 0.01
T-1249 + POPC/Chol bottom	0.08 ± 0.01	0.13 ± 0.01	27.50 ± 0.01

**Table 4:** Lennard-Jones (LJ) and Coulomb interaction energies between the peptides’ amphipathic domains, tryptophan-rich domain (TRD) and pocket binding domain (PBD), and the membrane lipids (POPC and Chol). The results are averaged over all the 100 ns of the simulations, so as to encompass all the aspects of the peptides’ behaviors. In the sums columns (∑) are totalized the interaction energies (LJ or Coulomb) of the membranes with both domains.

System		LJ energy (kJ mol^−1^)			Coulomb energy (kJ mol^−1^)		
	
		*TRD*	*PBD*	*∑*	*TRD*	*PBD*	∑
T-20 + POPC	POPC	−292.99 ± 0.88		−293	−146.23 ± 0.47		−146

T-1249 + POPC	POPC	−390.22 ± 1.13	−245.99 ± 0.99	−636	−260.99 ± 0.83	−223.08 ± 0.96	−484

T-20 + POPC/Chol	POPC	−296.48 ± 1.26		−311	−179.00 ± 0.96		−181
	Chol	−14.49 ± 0.11			−2.08 ± 0.03		

T-1249 + POPC/Chol	POPC	−177.83 ± 0.97	−158.51 ± 0.70	−350	−92.85 ± 0.65	−83.24 ± 0.54	−180
	Chol	−2.07 ± 0.02	−11.22 ± 0.08		−0.01 ± 0.01	−4.05 ± 0.07	

**Table 5:** Lateral diffusion coefficients for membrane lipids.

System	*D*_lat_ (10^−8^ cm^2^ s^−1^)	

	Chol	POPC
POPC		6.77 ± 0.07
POPC/Chol	0.33 ± 0.01	0.59 ± 0.04
T-20 + POPC top		5.29 ± 0.12
T-20 + POPC bottom		6.17 ± 0.13
T-20 + POPC/Chol top	0.60 ± 0.03	0.66 ± 0.03
T-20 + POPC:Chol bottom	0.61 ± 0.03	0.59 ± 0.03
T-1249 + POPC top		3.92 ± 0.14
T-1249 + POPC bottom		5.37 ± 0.19
T-1249 + POPC/Chol top	0.42 ± 0.03	0.41 ± 0.03
T-1249 + POPC/Chol bottom	0.64 ± 0.03	0.65 ± 0.04

**Table 6:** Average −S_CD_ for *sn-1* acyl chains in all systems.

System	−*S*_CD_*sn*-1	% variation
POPC	0.172 ± 0.010	
T-20 + POPC	0.176 ± 0.009	2.3
T-1249 + POPC	0.160 ± 0.009	−7.0
POPC/Chol	0.382 ± 0.017	
T-20 + POPC/Chol	0.368 ± 0.019	−3.7
T-1249 + POPC/Chol	0.356 ± 0.019	−6.8

**Table 7:** −*S*_CD_(*R*) for *sn-*1 acyl chains. *R* is the minimal distance between the peptide domain (TRD or PBD) and each individual POPC molecule.

System	Domain	Bin	*−S*_CD_*sn*-1	%difference *sn*-1
POPC			0.172 ± 0.010	

T-20 + POPC	TRD	*R* < 0.6 nm	0.080 ± 0.005	−53.5
		0.6 < *R* < 1.2 nm	0.157 ± 0.011	−8.9
		*R* > 1.2 nm	0.180 ± 0.010	4.6

T-1249 + POPC	TRD	*R* < 0.6 nm	0.103 ± 0.008	−40.2
		0.6 < *R* < 1.2 nm	0.165 ± 0.009	−3.8
		*R* > 1.2 nm	0.169 ± 0.010	−1.8

	PBD	*R* < 0.6 nm	0.171 ± 0.010	−0.4
		0.6 < *R* < 1.2 nm	0.174 ± 0.009	0.9
		*R* > 1.2 nm	0.155 ± 0.009	−9.9

POPC/Chol			0.382 ± 0.017	

T-20 + POPC/Chol	TRD	*R* < 0.6 nm	0.357 ± 0.017	−6.5
		0.6 < *R* < 1.2 nm	0.365 ± 0.019	−4.5
		*R* > 1.2 nm	0.370 ± 0.019	−3.0

T-1249 + POPC/Chol	TRD	*R* < 0.6 nm	0.358 ± 0.019	−6.4
		0.6 < *R* < 1.2 nm	0.354 ± 0.019	−7.5
		*R* > 1.2 nm	0.356 ± 0.019	−6.7

	PBD	*R* < 0.6 nm	0.356 ± 0.018	−6.7
		0.6 < *R* < 1.2 nm	0.370 ± 0.019	−3.1
		*R* > 1.2 nm	0.354 ± 0.019	−7.3
